# Supplementary material for: A Deep Learning-Based Model for Predicting Abnormal Liver Function in Workers in the Automotive Manufacturing Industry: A Cross-Sectional Survey in Chongqing, China
Source: Int J Environ Res Public Health. 2022 Nov 1;19(21):14300. doi: 10.3390/ijerph192114300 (PMC9655771; doi:10.3390/ijerph192114300)
Supplement: Supplementary file 1 [file ijerph-19-14300-s001.zip › ijerph-1978217-supplementary.pdf]

International Journal of
Environmental Research and Public Health

MDPI

SUPPLEMENTARY MATERIAL

Table S1. Final parameter values of the models.

Model	Parameter_name	Parameter_value
DNN	Epoch	1
	Number of hidden layers	3
	Number of neurons in the 1th hidden layer	3
	Number of neurons in the 2th hidden layer	3
	Number of neurons in the 3th hidden layer	3
XGBoost	Gamma	0
	Min_child_weight	1
	Nrounds	150
	Max_depth	3
	Eta	0.4
	Subsample	0.75
	Colsample_bytree	0.8
	Rate_drop	0.01
	Skip_drop	0.95
SVM	Kernel	Radial
	Sigma	0.09615234
	Cost	0.25
